# The Use of Microfiltration for the Pretreatment of Backwash Water from Sand Filters

**DOI:** 10.3390/ma17122819

**Published:** 2024-06-10

**Authors:** Małgorzata Wolska, Małgorzata Kabsch-Korbutowicz, Agata Rosińska, Anna Solipiwko-Pieścik, Halina Urbańska-Kozłowska

**Affiliations:** 1 Faculty of Environmental Engineering, Wroclaw University of Science and Technology, 27 Wybrzeże Wyspiańskiego st., 50-370 Wrocław, Poland; malgorzata.kabsch-korbutowicz@pwr.edu.pl (M.K.-K.); anna.solipiwko-piescik@pwr.edu.pl (A.S.-P.); 2 Faculty of Infrastructure and Environment, Czestochowa University of Technology, 60a Brzeźnicka st., 42-200 Czestochowa, Poland; agata.rosinska@pcz.pl; 3 Wroclaw Municipal Water and Sewage Company, Na Grobli 14/16, 50-421 Wrocław, Poland; halina.urbanska@mpwik.wroc.pl

**Keywords:** microfiltration process, psychrophilic bacteria, recirculation, water treatment plants

## Abstract

Tests of microfiltration efficiency used for the pretreatment of backwash water from sand filters were conducted at two water treatment plants treating surface water and infiltration water. Microfiltration efficiency was evaluated for three membrane modules: two with polymeric membranes and one with a ceramic membrane. This study showed that the contaminants that limit the reuse of backwash water from both plants by returning them to the water treatment line are mostly microorganisms, including pathogenic species (*Clostridium perfringens*). Additionally, in the case of backwash water from infiltration water treatment, iron and manganese compounds also had to be removed before its recirculation to the water treatment system. Unexpectedly, organic carbon concentrations in both types of backwash water were similar to those present in intake waters. Microfiltration provided for the removal of organic matter, ranging from 19.9% to 44.5% and from 7.2% to 53.9% for backwash water from the treatments of surface water and infiltration water, respectively. Furthermore, the efficiency of the iron removal from backwash water from infiltration water treatment was sufficient to ensure good intake water quality. On the other hand, manganese concentrations in the backwash water, from infiltration water treatment, pretreated using the microfiltration process exceeded the levels found in the intake water and were, therefore, an additional limiting factor for the reuse of the backwash water. In both types of backwash water, the number of microorganisms, including *Clostridium perfringens* (a pathogenic one), was a limiting parameter for backwash water reuse without pretreatment. The results of the present study showed the possibility for using microfiltration for the pretreatment of backwash water, regardless of its origin but not as the sole process. More complex technological systems are needed before recirculating backwash water into the water treatment system. The polyvinylidene fluoride (PVDF) membrane proved to be the most effective for DOC and microorganism removal from backwash water.

## 1. Introduction

More and more regions of the world suffer from water deficits and water stress, resulting in limited access to water for more and more people [1]. This means there is a need to search for new sources of water and reduce water losses by introducing a circular economy and the sustainable management of water resources [2]. Roughly 12% of the world’s water is consumed in households, with rapid filtration processes resulting in backwash water that accounts for between 2% and 10% of the plant’s capacity [3]. Therefore, the wastewater stream is the subject of research on the possibility of its reuse. In water supply systems, this wastewater is the most often discharged into sewage systems or the soil, while it can be an additional potential source of water. The rising costs of water intake and wastewater disposal are driving research on the feasibility for reusing backwash water, e.g., from land irrigation or recirculation to water treatment systems [4]. One of the unconventional applications of filter backwash water is its reuse as a coagulant for wastewater treatment [5]. However, the pretreatment of the backwash water is required for each type of reuse, and the types of processes used depend on the backwash water’s origin and composition. Furthermore, the composition of the backwash water is determined by the filtration parameters, the type and quality of the water being treated, and the type and granulation of the filter beds [6]. In the case of backwash water generated during surface water treatment, the parameters limiting the backwash water’s return to the water treatment system include a very high content of microorganisms and organic substances [7]. At water treatment plants, the backwash water generated during surface water treatment contains total organic carbon in the range 8.5–60.0 gC/m^3^, whereas in raw water, its concentration is from 2.1 to 5.0 gC/m^3^ [8]. At the WTP in Isfahan, raw backwash water contains, on average, 9500 coli group bacteria [9].

In the cases of groundwater and infiltration water, the highest pollutant load is observed in iron and manganese compounds, up to 1500 mg/dm^3^ and 20 mg/dm^3^, respectively [10], with their removal requiring a complex backwash water pretreatment system [6]. This backwash water may also contain microorganisms, including pathogenic species, thus making them difficult to reuse.

Conventional processes, such as coagulation and sedimentation, are the most commonly used to pretreat filter backwash water [11], but low-pressure membrane separation processes are also increasingly applied to provide for greater pretreatment efficiency [12,13,14,15]. Pressurize-driven membrane processes are now very widely used to treat water from various sources for both drinking and industrial purposes [16].

The effectiveness of microfiltration membranes in wastewater treatment depends on the type of membrane, pore size, composition of the medium to be treated, and process conditions [17]. Despite the implementation of microfiltration in water and wastewater treatments, including industrial wastewater, there is no information about its efficiency in sand-filter backwash water pretreatment. Generally, microfiltration is currently used for particle and microorganism removal [18] from both types of backwash waters.

Therefore, it was warranted to explore the possibilities for treating backwash water from surface and infiltration water treatment plants using microfiltration. Therefore, the purposes of this study were to reach the quality level of the intake water and to assess the suitability of different types of microfiltration membranes. The results obtained will add to the knowledge of the possibility of the use of microfiltration in backwash water treatment with different compositions.

## 2. Materials and Methods

**Figure 1:**
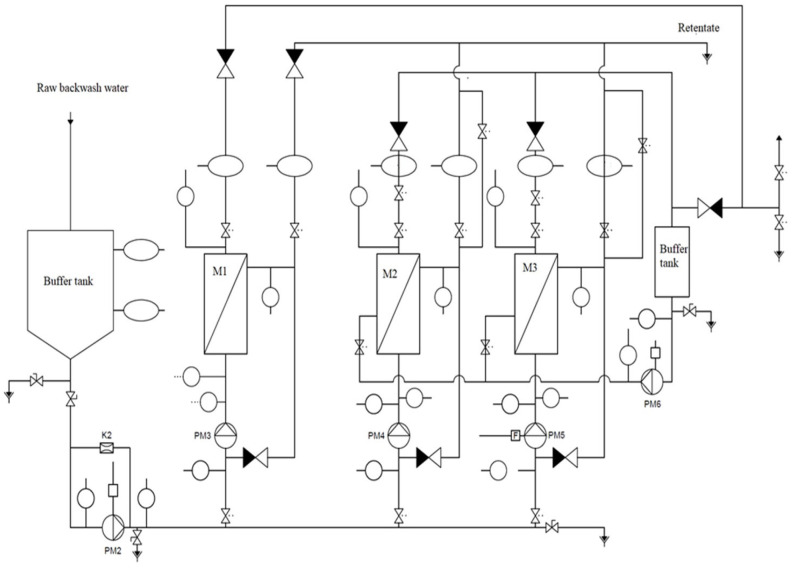
Microfiltration test procedure (M1, M2, and M3—membranes; PM2, PM3, PM4, PM5, and PM6—pumps; K2—sensor).

The research on the efficiency of the pretreatment of backwash water using sand filters was conducted at two water treatment plants in southwest Poland treating surface and infiltration waters. At both plants, the backwash water generated during water treatment is collected in sedimentation tanks, where they undergo preliminary sedimentation. The backwash water, after the initial sedimentation (for 8 h in existing tanks), was subjected to microfiltration tests in a pilot flow installation, as shown in Figure 1. Backwash water from water treatment plants was collected in settling tanks and, after 8 h of sedimentation, supplied to the microfiltration installation (buffer tank) (Figure 1). The pretreatment system included bag filters with a pore size of 20 µm. For the filter materials used, technical data were not made available. The stability of the backwash water flow was ensured by a buffer tank, which was the first element of the research installation.

**Figure 2:**
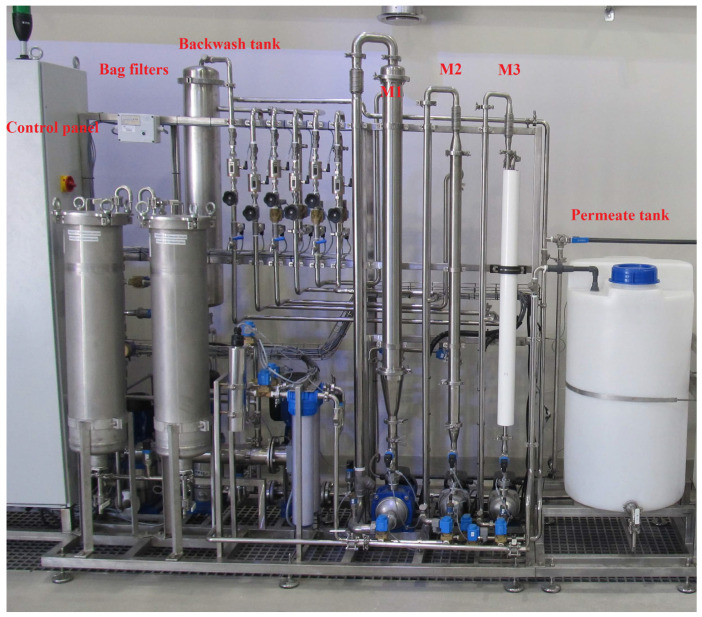
Microfiltration plant.

The installation features three membrane module housings to test capillary and spiral membranes (Figure 2). The system is equipped with automatic backwashing of the capillary modules, which takes 30 s and is repeated every 10 min. However, because of the specific design of the installation, the spiral module was not backwashed. Tests were conducted for three membrane modules: spiral with a polymer membrane (M1), capillary with a polymer membrane (M2), and capillary with a ceramic membrane (M3). Three tests lasting 7 days were carried out for each membrane. Because of the significant effect of the temperature on the membrane separation process, the tests were conducted at different times of the year. Samples of pretreated backwash water were collected after 2 h, 24 h, 2 d, 3 d, and 7 d in each test series. The characteristics of the membrane modules used in the tests are presented in Table 1.

Microbiological analyses were performed using seeding methods in accordance with applicable standards: PN-EN ISO 6222:2004—the total number of microorganisms, PN-EN ISO 9308-2:2014-06—coliform bacteria and *Escherichia coli*, PN-EN ISO 7899-2: 2004—*Enterococci*, and PN-EN ISO 14189:2016-10—*Clostridium perfringens*.

Regardless of the membrane used and the origin of the backwash water, a transmembrane pressure of 2.5 bar was used in all the tests. The capacity of the microfiltration installation was 100 dm^3^/h for every membrane module. The pressure, flow rate, and temperature were measured online and recorded every 10 min. After each test, the membranes were chemically cleaned with a 2% citric acid solution and a 15% sodium hypochlorite solution.

Raw backwash water (after the initial sedimentation in settling tanks and after the microfiltration process) was analyzed for its physical and chemical compositions, including pH, color, turbidity, UV_254_ absorbance, concentration of dissolved organic carbon (DOC), and microbiological content, including the total number of psychrophilic microorganisms and the number of bacteria from coliforms, *Escherichia coli*, *Enterococci*, and *Clostridium perfringens*. The molecular weight distribution of the organic particles in the feed water and in raw backwash water was measured by the chromatographic method using an UltiMate 3000 Dionex liquid chromatograph (Dionex, Sunnyvale, CA, USA) equipped with a DAD detector (Dionex, Sunnyvale, CA, USA).

Measurements of pH were performed using a Hach HQ440D multi-parameter meter(Hach, Loveland, CO, USA); turbidity was measured using a Hach TU5200 turbidimeter (Hach, Loveland, CO, USA), and the concentration of the DOC was determined using a Shimadzu TOC-L TOC analyzer (Shimadzu, Kioto, Japan). Color and absorbance measurements were performed in samples filtered through 0.45 µm filters using a Shimadzu UV-Vis UV-1200 spectrophotometer (Shimadzu, Kioto, Japan).

## 3. Results and Discussion

The recycling of the pretreated backwash water into the water treatment system is justified if it does not constitute a health hazard and does not negatively affect other processes. Therefore, in the present research, it was assumed that the pretreatment of the backwash water should ensure a quality that is at least comparable to the composition of the intake water.

As previous studies have shown [19], backwash water from surface water treatment had greater variability in the composition over the year. Irrespective of the type of backwash water, the presence of microorganisms and organic substances, and, in the case of the microfiltration of backwash water from infiltration water treatment, the contents of iron and manganese compounds pose the greatest problems with backwash water’s reuse.

**Figure 3:**
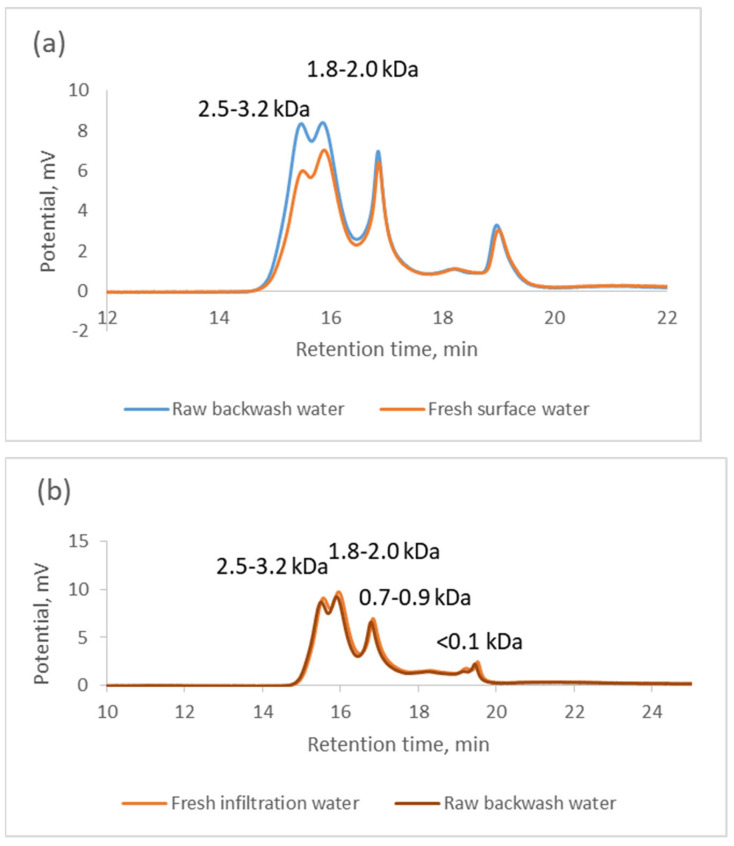
Particle size distributions in the intake water and raw backwash water: (**a**) surface water, (**b**) infiltration water; KDa—kilodalton.

As shown in Table 2, the backwash water from the infiltration water treatment plant contained slightly higher amounts of organic matter than that from surface water treatment, whereas the variability of its concentration was lower than that in the backwash water from surface water treatment. Among the organic substances, compounds with low molecular weights were predominant in the raw and microfiltration backwash waters, as evidenced both by the specific UV_254_ absorbance values, which were slightly higher in the backwash water from infiltration water treatment, and by the results of the analysis of the particle size distribution (Figure 3). Particles of organic matter, determined in the intake water, in the backwash water were on the order of a few nanometers in size [20]. The literature data show that the smaller the particle size of the filtered fraction, the greater the observed decreases in the flow rate and filtration resistance. Particles with low molecular weights can easily enter and adsorb on membrane pores and cause membrane fouling, while those with higher weights cause gel formation or concentration polarization, which prevents small particles from entering the membrane pores. Undissolved organic matter (in water after filtration through a 1 μm filter), which is much larger than the pores of UF and MF membranes, has been found to form a coarse cake that does not increase fouling. In contrast, dissolved organic matter (in water after filtration through a 0.45 μm filter) forms a dense gel on the membrane surface and blocks the pores, thus playing a significant role in fouling [21].

**Figure 4:**
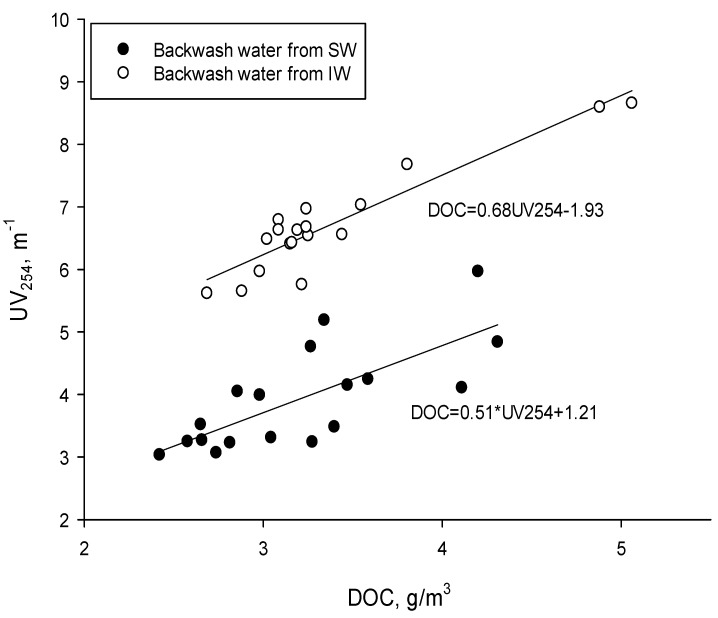
Relationship between DOC concentration and UV254 absorbance for backwash water before and after microfiltration (regression equations for IW, DOC = 0.6802UV_254_ − 1.1929, and SW, DOC = 0.5077UV_254_ + 1.2097).

In both types of backwash water, the UV_254_ absorbance was proportional to the dissolved organic carbon content (Figure 4), indicating the presence of refractive compounds [22]. This relationship was found for both raw backwash water and that after microfiltration, indicating that refractory compounds were mainly retained in this process.

The content of the dissolved organic matter in both types of raw backwash water was higher than permissible levels in drinking water and slightly higher than that found in the raw intake waters (Table 2). High concentrations of iron and manganese were also found in the backwash water from filters used for infiltration water treatment. This indicates that both types of backwash water needed to be pretreated before being recirculated into the technological systems of water treatment.

The present research showed that the efficiency of the pretreatment of backwash water using the microfiltration process depends primarily on the properties of the membranes used and the composition of the backwash water used in the process (Table 3).

The retention rates of the dissolved organic matter in both types of backwash water ranged from 9.2 to 53.9% and depended on the type of membrane. The high retention of DOC in MF membranes with nominal pores of 0.2 μm is surprising and may result, among other things, from the formation of a filter cake on the membrane surface, which represents an additional dynamic membrane. The same observation was reported by Gray et al. [23], with a retention rate of DOC greater than that found in other studies on the removal of organic matter using microfiltration of drinking water [24,25]. According to Wakeman [26], during filtration, the finest particles control many aspects of the process cycle. The smallest particles are those that pass through the filter membrane at the initial stages of filtration, accumulate in the cake layers the closest to the filter membrane, contribute the most to the specific surface area of the particles and the specific resistance of the filter cake, and interact the most strongly with ions or other substances (for example, polymers) in the solution, creating a compressibility effect. Particle size is the most important for determining filtration, with finer particles in the distribution usually ‘controlling’ filtration. Even a small increase in the number of these finer particles can significantly reduce the filtration rate [26].

**Figure 5:**
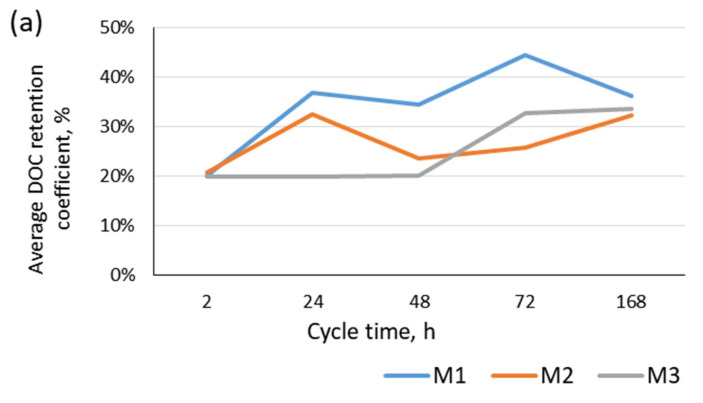

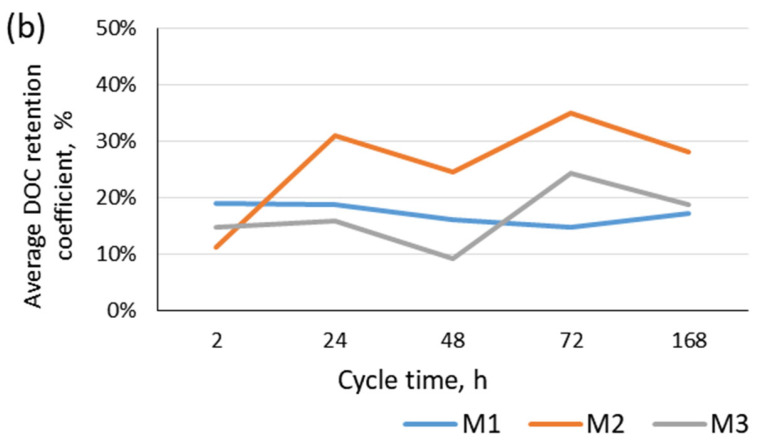
Effect of microfiltration time on the degree of reduction in dissolved organic carbon concentration: (**a**) backwash water from surface water treatment; (**b**) backwash water from infiltration water treatment.

In the case of backwash water from surface water treatment, the membrane mounted in the module with the spiral configuration (M1), which was not backwashed, proved to be the most effective. This was probably because of the formation of a dynamic membrane layer (of retained contaminants) on the membrane surface, allowing for the retention of substances with lower molecular weights [20]. This mechanism may confirm the observed increase in the retention rate up to the 72nd hour of the cycle, after which the membrane fouling was already intense enough to lead to a reduction in organic matter retention. In contrast, no such relationship was found for the other two backwashed membrane modules (Figure 5a).

In contrast, in the case of the treatment of backwash water from the infiltration water treatment plant, the membrane in the M1 module was characterized by a stable level of organic matter retention throughout the filtration cycle (Figure 4). The lower organic retention efficiency for this membrane can be explained by the presence of large amounts of iron and manganese compounds, with their presence intensifying membrane fouling. For the other two membranes, the degree of organic matter removal was similar to that found for backwash water from surface water treatment because of the cyclic mechanical removal of contaminants from the membrane surface using backwashing (Figure 5b).

With the efficiency of the removal of organic matter, its concentration was reduced to a level below that observed in the intake water, which allows for the backwash water from the microfiltration process to be returned to the initial part of the technological system for the treatment of both surface and infiltration waters.

The retention rates of iron and manganese compounds (Table 4) indicated very effective removal of iron compounds, regardless of the type of membrane used.

**Figure 6:**
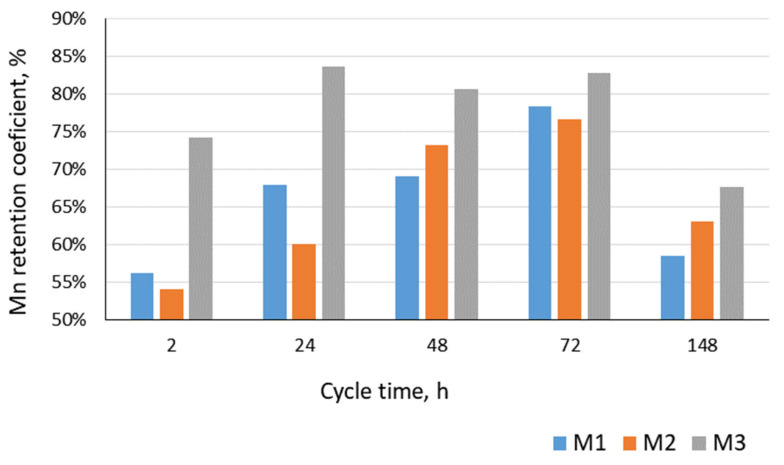
Average values of manganese retention factors in the microfiltration process of infiltration backwash water treatment.

In contrast, manganese compounds were removed with lower efficiencies, regardless of the cycle time. The highest retention of manganese was found for the ceramic membrane (Figure 6), while the other two were characterized by a lower and similar degree of removal of these contaminants. At the same time, none of the membranes used ensured the removal of manganese compounds to the levels found in the infiltration water. Furthermore, the efficiency of the manganese removal was similar to that documented by Lin et al. [27] during the microfiltration of water containing iron and manganese compounds using a PP membrane. These researchers also demonstrated a reduction in the manganese removal efficiency in the presence of iron in the water. On the other hand, the oxidized manganese present in the treated solution caused severe membrane fouling during the crossflow and dead-end membrane separation, and the manganese dioxide aggregate was prone for membrane fouling [28] as a consequence of the efficiency of the manganese removal decreasing with the increase in the manganese dioxide concentration.

**Figure 7:**
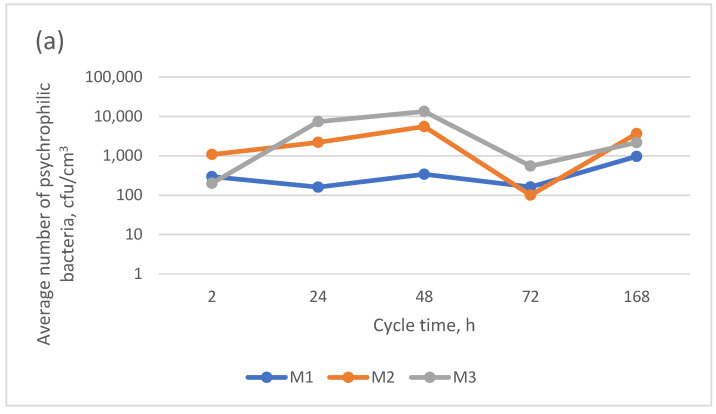

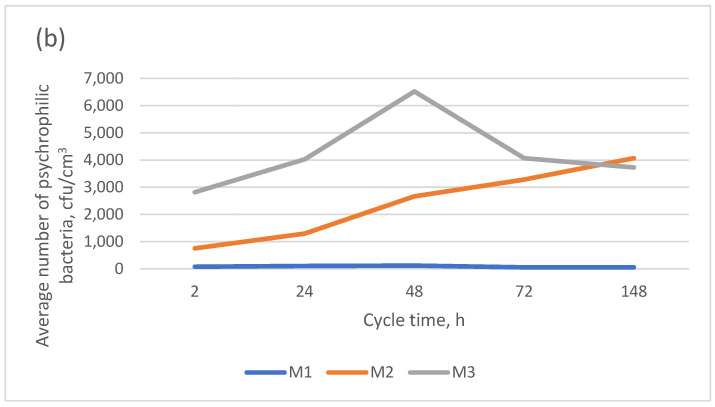
Average numbers of psychrophilic bacteria in microfiltration permeate: (**a**) backwash water from surface water; (**b**) backwash from infiltration water.

The backwash water from surface water treatment was characterized by a very high psychrophilic bacteria count (Table 2), between 1.5 and 12 times higher than that found in the backwash water from infiltration water treatment. The presence of such a large number of microorganisms significantly affected their counts in the permeate after microfiltration (Figure 7).

Regardless of the type of backwash water, the highest retention rate was achieved for the PVDF membrane (installed in the spiral module), with its values similar to that obtained for the 0.1 µm pore membrane made of the same material used in surface water treatment [25]. The high efficiencies of the elimination of psychrophilic bacteria, ranging from 90.8 to 98.6% and from 98.5 to 99.2% for backwash water from the treatments of surface water and infiltration water, respectively, may have been caused by the formation of a filter cake on the membrane surface, which was not effectively eliminated because of the lack of backwashing in this module [29]. It provides an additional membrane to effectively retain psychrophilic bacteria.

As shown by the analytical results of this study, the psychrophilic bacteria count in the M1-module-treated backwash water from surface water treatment was similar to or greater than that found in the intake surface water, which may have a negative impact on the effectiveness of the treatment when the treated backwash water is recirculated into the technological system. The return of these pretreated backwash waters would require an additional disinfection process before their combination with the intake water stream.

In all the samples of backwash water from infiltration water treatment using the M1 module, the psychrophilic bacteria count was lower than that found in the intake infiltration water, allowing for this backwash water to be returned to the water treatment system.

Irrespective of the type of backwash water being treated, the membranes in modules M2 and M3 did not provide for sufficient removal of these bacteria to allow for the treated backwash water to be returned to the water treatment system without disinfection. Chen et al. [28] showed that during filter cake formation, the size of the removed particles is smaller, and, as a consequence, the efficiency of the bacterial retention was the highest for the membrane in module 1, which was not backwashed throughout the operation time.

**Figure 8:**
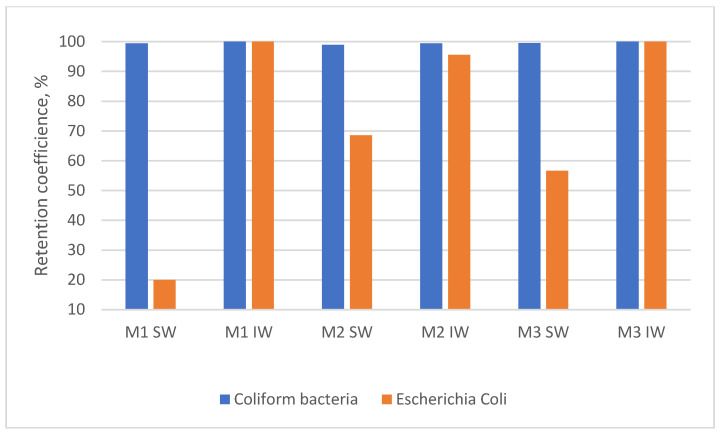
Average retention rates of indicator microorganisms.

The inability to return the backwash water from surface water treatment without using additional disinfection is also evidenced by the insufficient retention of indicator bacteria, especially *E. coli* (Figure 8).

In general, irrespective of the type of membrane used, the efficiency of the removal of indicator bacteria from backwash water from surface water treatment was lower than that found for backwash water from infiltration water treatment. The presence of indicator microorganisms in treated backwash water may pose a health risk if they are returned to water treatment systems [30]. For all three membranes, irrespective of the type of backwash water, the lowest removal efficiency was achieved for *E. coli*.

As shown by the results, the efficiency of the microfiltration in the pretreatment of backwash water generated both during the operation of rapid filters used to treat surface water and infiltration water, regardless of the type of membrane, is sufficient to return the backwash water in terms of the presence of organic substances. Furthermore, as found in other studies [31], none of the membrane modules ensured the biological stability of the pretreated backwash water. The greatest efficiency of the removal of microorganisms from backwash water from both plants was achieved using the M1 module. The use of this module also ensured the greatest removal of indicator microorganisms from backwash water from infiltration water treatment. In the case of backwash water from surface water treatment, greater efficiency was obtained for indicator bacteria by M2 and M3.

The use of a PVDF membrane (M1 module) for the pretreatment of backwash water from infiltration water treatment is not recommended because of insufficient efficiency in removing manganese. Returning this backwash water to the technological system requires additional pretreatments for manganese removal and biological stabilization. Such an elaborate technological system is not economically feasible, and this points to the need for a different backwash water pretreatment process, such as ultrafiltration.

At the same time, in the case of backwash water from surface water treatment, none of the membranes tested provided for the sufficient elimination of microorganisms to return microfiltration-treated backwash water, even to the initial part of the technological system.

**Figure 9:**
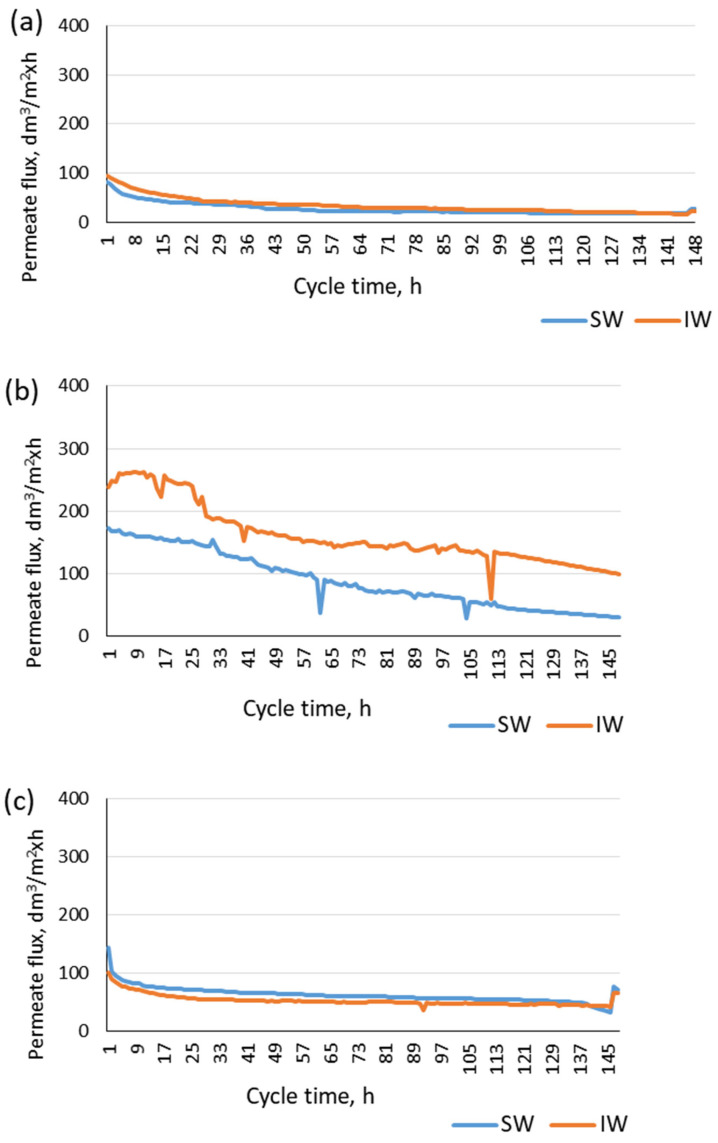
Changes in average permeate flux for modules (**a**) M1, (**b**) M2, and (**c**) M3.

Because of the different properties of the membranes (different materials: M1—PVDF; M2—PP; M3—ceramic membrane) in the modules used for the pretreatment of backwash water from water treatment and as a result of the different configurations of the membrane modules (spiral module M1 and capillary modules M2 and M3), a different permeate flux was recorded for each module. Regardless of the origin of the backwash water (surface water or infiltration water), the lowest permeate flux was obtained for the spiral module (M1), with a membrane made of PVDF, which was not backwashed during microfiltration. This is consistent with the results presented by Yang et al. [32], who found that the frequency of the backwashing significantly affects the reduction in PVDF membrane fouling. Furthermore, a slightly higher permeate flux was obtained for this membrane when treating backwash water from infiltration water treatment (Figure 9a).

Similarly, for the membrane made of polypropylene (module M2) (Figure 9b), the permeate flux was higher for infiltration water treatment and nearly twice as high as that found for module M1 (Figure 9a). Irrespective of the type of backwash water, the highest permeate flux was obtained for the M3 module with a ceramic membrane (Figure 9c), which is consistent with literature reports [33].

In the case of all the membrane modules, a decrease in the permeate flux was observed, irrespective of the type of backwash water to be treated and the way the module was operated (with or without membrane backwashing). As illustrated in Table 5, the relative permeabilities of the membranes, defined as the ratio of the permeate flux after 7 days of module operation to the flux at the beginning of the microfiltration cycle, were 0.33, 0.17, and 0.49 for modules M1, M2, and M3, respectively, for the microfiltration of backwash water from surface water treatment. In the case of backwash water from infiltration water treatment, the decreases were 0.25, 0.41, and 0.64, respectively. This indicates that the ceramic membrane was the least susceptible for fouling, irrespective of the type of backwash water. At the same time, the present study also found that, of the polymeric membranes, more intensive fouling was observed for the M2 module (PP membrane) when filtering backwash water from surface water treatment and for the M1 module (PVDF membrane) when filtering backwash water from infiltration water treatment. Gray et al. [23] showed that membranes made of PVDF are less susceptible for organic-induced fouling than membranes made of PP.

Despite the same pore size of the membranes installed in each module, the variation in the reduction in the permeate flux is because of the different material properties of the individual membranes, having a significant effect on the intensity of the fouling [34]. Significant differences in the permeate flux, depending on the type of backwash water used for microfiltration, were found only for the PP membrane. This means that the membrane material affects the permeate flux achieved to a greater extent than using membrane backwashing.

## 4. Conclusions

The results of this study showed differences in the removal efficiencies of individual contaminants, depending on the type of backwash water. It should be noted that the concentration of manganese in backwash water from infiltration filters remains a problem that can hinder the reuse of this water. Therefore, additional manganese removal methods may be necessary to ensure that high-quality water is returned to the water treatment process. It is also worth noting that microfiltration can be an effective tool in the treatment of backwash water, but it is necessary to continue research on optimizing this process to ensure the efficient and economical operation of water treatment plants.

The main findings of this study are as follows:

1. The composition of the backwash water subjected to microfiltration has the greatest effect on the pretreatment efficiency;
2. All the types of microfiltration membranes used allowed for the effective and sufficient returning of treated backwash water to the main water treatment line and the removal of organic substances in ranges from 19.9% to 44.5% and from 7.2% to 53.9% from backwash water from the treatments of surface water and infiltration water;
3. Regardless of the type, none of the microfiltration membranes used ensured the biological stability of the pretreated backwash water;
4. The average numbers of psychrophilic bacteria in the permeate were 384, 3625, and 2563 from M1, M2, and M3, respectively; the PVDF microfiltration membrane had the highest psychrotrophic bacterial retention rate;
5. None of the microfiltration membranes used ensured the removal of manganese compounds to the levels found in the infiltration water. Their lowest concentrations were found in permeate from M3 (average: 125 µg/dm^3^);
6. The PVDF membrane showed the highest permeate flux stability, whereas the PP membrane was characterized by the lowest stability;
7. Microfiltration cannot be used as the sole process for the pretreatment of back-wash water before it is returned to the water treatment system, regardless of its origin.

## Figures and Tables

**Table 1:** Characteristics of the membrane modules used.

Technical Data	Membrane Module		
	M1	M2	M3
Manufacturer/supplier	Polymem Tech		
Module type	spiral	capillary	capillary
Membrane material	Poly vinylidene fluoride (PVDF)	Polypropylene (PP)	α Alumina
Maximum permissible temperature (°C):	45	45	140
Maximum allowable pressure (MPa):	1.0	0.3	0.8
Membrane (pore size) (μm):	0.2	0.2	0.2
Membrane (m^2^):	1.8	0.5	1.26

**Table 2:** Characterization of raw backwash water.

Parameter	Unit	Surface Water		Infiltration Water	
		Fresh Water	Raw Backwash Water	Fresh Water	Raw Backwash Water
Turbidity	NTU	1.3–22	1.2–3.9	4.2–37	59–92
Color	g/m^3^	1–38	3–5	6.0–28	7–10
pH		7.1–8.2	7.2–8.7	6.8–7.8	7.4–7.6
UV_254_	m^−1^	6.3–27.2	3.99–7.49	6.3–11.4	6.60–9.77
SUVA	m^2^/g		0.93–1.66		1.47–2.16
DOC	g/m^3^	3.07–10.2	3.32–5.20	2.91–5.9	3.45–6.62
*Enterococci*	cfu/100 cm^3^	0–100	0–28	0–15	0–3
*Clostridium perfringens*	cfu/100 cm^3^	7–290	1–10	0	0
Coliform bacteria	cfu/100 cm^3^	2–9200	55–2420	0–201	8–276
*Escherichia Coli*	cfu/100 cm^3^	1–790	1–5	0	2–5
Psychrophilic bacteria	cfu/1 cm^3^	490–170,000	6200–210,000	11–550	4700–18,000
Fe	g/m^3^	0.06–0.64	0.02–0.52	0.61–5.59	6.35–12.11
Mn	g/m^3^	0.01–0.29	0.02–0.25	0.25–0.55	0.35–0.58

**Table 3:** Characterization of backwash water after microfiltration.

Parameter	Unit	Surface Water			Infiltration Water		
		M1	M2	M3	M1	M2	M3
Turbidity	NTU	0.3–0.32	0.3–0.36	0.3–1.1	0.3	0.3–1.7	0.3
Color	g/m^3^	2–4	2–6	2–4	7–8	7–11	5–8
pH		7.8–8.7	7.9–8.4	6.8–8.6	7.3–7.7	7.5–7.7	7.5–7.7
UV_254_	m^−1^	2.89–3.45	2.91–5.58	2.62–4.69	6.36–7.20	6.21–13.3	4.91–6.17
SUVA	m^2^/g	0.82–1.38	1.16–1.51	0.91–1.43	1.99–2.28	1.29–2.08	1.62–2.35
DOC	g/m^3^	2.22–3.65	2.35–4.8	2.50–4.25	3.01–3.20	3.05–5.31	2.46–3.22
*Enterococci*	cfu/100 cm^3^	0	0	0	0	0	0
*Clostridium perfringens*	cfu/100 cm^3^	0	0	0	0	0	0
Coliform bacteria	cfu/100 cm^3^	1	1	0–1	0	0–1	0
*Escherichia Coli*	cfu/100 cm^3^	1	1–5	0–1	0	0–2	0
Psychrophilic bacteria	cfu/1 cm^3^	4–1600	200–5900	2–40,000	8–230	8–8000	39–13,000
Fe	g/m^3^	0.02	0.02–0.05	0.02–0.21	0.02–0.06	0.02–0.63	0.02
Mn	g/m^3^	0.005	0.01–0.02	0.01–0.12	0.07–0.30	0.04–0.25	0.06–0.23

**Table 4:** Ranges of contaminant retention factors.

Parameter	Surface Water						Infiltration Water					
	M1		M2		M3		M1		M2		M3	
	min.	max.	min.	max.	min.	max.	min.	max.	min.	max.	min.	max.
Turbidity	83.7%	87.1%	88.5%	96.2%	74.5%	95.7%	99.5%	99.5%	98.4%	99.6%	99.6%	99.6%
Color	0.0%	31.7%	0.0%	22.5%	8.3%	33.3%	0.0%	12.5%	5.0%	30.0%	5.6%	27.0%
UV_254_	19.3%	27.2%	10.3%	32.5%	14.1%	35.1%	9.2%	16.4%	0.7%	36.4%	8.1%	20.4%
DOC	20.1%	44.5%	20.7%	32.5%	19.9%	33.5%	14.7%	18.9%	7.8%	53.9%	9.2%	24.4%
Psychrophilic bacteria	90.8%	98.6%	10.0%	99.9%	23.7%	98.1%	98.5%	99.3%	77.1%	99.9%	59.1%	71.6%
Fe	71.0%	74.2%	31.0%	87.5%	4.8%	33.7%	99.3%	99.7%	96.5%	99.8%	99.8%	99.8%
Mn	66.9%	74.8%	79.6%	84.0%	56.4%	77.8%	56.1%	78.4%	43.5%	76.7%	67.6%	83.7%

**Table 5:** Changes in membrane transport properties during pretreatment of filtration backwash water.

Module	Initial Permeate Flux, dm^3^/m^2^h		Relative Membrane Permeability (after 7 Days of Use)	
	Surface Water	Infiltration Water	Surface Water	Infiltration Water
M1	82.30	95.15	0.33	0.25
M2	173.06	237.25	0.17	0.41
M3	143.34	100.50	0.49	0.64

## Data Availability

Data are contained within the article.
